# Microtubule-associated protein 1B is implicated in stem cell commitment and nervous system regeneration in planarians

**DOI:** 10.1371/journal.pone.0278966

**Published:** 2022-12-12

**Authors:** Gaetana Gambino, Leonardo Rossi, Paola Iacopetti, Claudio Ghezzani, Patrizia Guidi, Stefania Linsalata, Chiara Ippolito, Alessandra Salvetti

**Affiliations:** 1 Department of Clinical and Experimental Medicine, University of Pisa, Pisa, Italy; 2 Medical Physics Unit, Azienda Ospedaliera Universitaria Pisana, Pisa, Italy; Pontificia Universidad Catolica de Chile, CHILE

## Abstract

Microtubule-associated 1B (MAP1B) proteins are expressed at the nervous system level where they control cytoskeleton activity and regulate neurotransmitter release. Here, we report about the identification of a planarian MAP1B factor (*DjMap1B*) that is enriched in cephalic ganglia and longitudinal nerve cords but not in neoblasts, the plentiful population of adult stem cells present in planarians, thanks to which these animals can continuously cell turnover and regenerate any lost body parts. *DjMap1B* knockdown induces morphological anomalies in the nervous system and affects neoblast commitment. Our data put forward a correlation between a MAP1B factor and stem cells and suggest a function of the nervous system in non-cell autonomous control of planarian stem cells.

## Introduction

Stem cells are unspecialized cells capable to self-renew and give rise to differentiated progenies. There are stem cells with various differentiation potentials i.e unipotent, multipotent, pluripotent and totipotent stem cells. In most organisms, pluripotent stem cells are only present during the early stages of embryogenesis, whereas multipotent and unipotent stem cells reside in adult tissues. An exception is represented by planarians (Platyhelminthes), aquatic invertebrates that have been always known for their great capability to regenerate any lost body fragment thank to the existence of a plentiful population of adult stem cells, the neoblasts, the only somatic proliferating cells in adults [1, 2]. After injury, molecular factors are released by the wound and a regenerative blastema, the region in which missing structures will differentiate, is produced by neoblast proliferation and accumulation [3]. Although neoblasts share similar morphology, they appear transcriptionally heterogeneous and different sub-populations expressing specific markers have been identified [1]. Among this heterogeneous population of cells, some neoblasts (c-neoblasts) have clonogenic activity being able to differentiate into all cell types when engrafted into lethally irradiated host, thus representing adult pluripotent stem cells [4]. Recently, the existence of a specific c-neoblast sub-population expressing exclusive genes has been questioned, and a single step fate model of specialization and potency has been proposed [5]. In this model, an undifferentiated neoblast in the G1-phase of the cell cycle, progressing through the cell cycle, becomes specialized starting to express specific tissue transcription factors. At the end of the cycle, in the case of asymmetric division, a newly produced neoblast could reenter the cell cycle and specialize towards a different cell fate suggesting that a specialized neoblast could reacquire pluripotency [5]. Additional evidence in support of such plasticity have been recently provided [6, 7].

Although several endogenous factors involved in neoblast regulation have been identified over the years, a fewer data are available about the role of external signals released by differentiated cells [8], despite the presence of diffusible signals recognized by neoblast receptors has been proposed [9–11]. Some indirect data suggest that intestine and nervous system might have a role in controlling neoblast proliferation and differentiation [8, 12]. Classical studies indicate that deletion of the nervous system interferes with the planarian regeneration capability and an increase and accumulation of neurosecretory granules have been found during the first hours of regeneration [13]. The role of neurotransmitters in neoblast biology is still an open question although it has been found that neuropeptide P and F, as well as substance K, promote neoblast proliferation, and somatostatin-like peptide reduces mitotic activity [9, 14–16].

An interesting model system for studying the influence of the nervous system on neoblast biology is represented by planarians irradiated with a low-dose of X-rays [12]. In this stressing condition, animals do not dye as the initial decline in neoblast number is compensated by an intense proliferation of some radioresistant cells situated very close to the ventral nerve cords, the so-called neoblast repopulation process [17]. Thus, it has been hypothesized that the cross-talk between neoblasts and surrounding differentiated tissues is particularly emphasized in low-dose irradiated animals, as they have to rapidly replenish the neoblast compartment. A possible role of the nervous system in this process has been also suggested by the evidence that spantide, an antagonist of the neuropeptide substance P, induces a reduction in the repopulation process [17]. Moreover, several genes transcribed in differentiated tissues, such as the nervous system and the gut, have been found up-regulated in low-dose irradiated planarians and their silencing by RNA interference (RNAi) affected neoblast repopulation process [18]. In this paper, we focus on a gene, *DjMap1B*, that is up-regulated in low-dose irradiated animals and we investigate its involvement in neoblast repopulation and its role in tissue regeneration and stem cell behavior.

## Material and methods

### Animals

The asexual GI strain (*Dugesia japonica*) [19] planarians were used. Animals were kept at 18°C in artificial medium [20], and famished for 2 weeks before being used in the experiments. *D*. *japonica* are invertebrates and all the testing were performed in accordance with Italian guidelines and recommendations.

### X-ray irradiation

Planarians were treated in single fraction with 15MV X-rays at the low-dose of 7 Gy or at the lethal dose of 30 Gy (dose rate 3 Gy/min or 6 Gy/min, respectively), by mean a Varian Medical System Clinac DHX linear accelerator for radiotherapy. The irradiation set-up (i.e. energy, field size, buildup thickness and material) was optimized in order to deliver a uniform radiation dose, within ± 2%, to the all specimen.

### *DjMap 1B* sequence identification

We identified a short sequence showing homology to Microtubule-associated protein 1B (and thus named: *DjMap1B*) in a DGE library including TAGs differentially expressed between planarians treated with the X-ray dose of 7 Gy and unirradiated animals (*DjMap 1B* overexpression fold change is 2,4 in low-dose X-ray treatment respect to untreated animals) [18]. Partial sequence was elongated by searching in the Planmine database [21] in which we identified the clone dd_Djap_v4_23650_3_1 encoding for a putative protein long 1247 aminoacids. The homolog in *S*. *mediterranea* Map1B was found by using Planmine web resource and joining clones sm_v6_3654_0_1, dd_Smes_g4_14 and sm_v6_1301_0_1 (blast 2: 76% nucleotide identity between the *DjMap1B* and *SmedMap1B*). Sequence homology with *Drosophila* and mouse has been found using BLASTx search tool.

### RNAi experiments

RNAi experiments were performed as described in [18]. Briefly, *DjMap1B* double-stranded RNA (dsRNA) was in vitro synthesized by using TranscriptAid T7 High Yield Transcription kit (Thermo Scientific) using the following primers:

*DjMap1B* T7 forward
5’ CGGATATAATACGACTCACTATAGGGATAGTATTCCTCCAAAACCATCA 3’
*DjMap1B* T7 reverse
5’ CGGATATAATACGACTCACTATAGGGGCATGGCATATTGTTGTCACC 3’

Animals were injected with 3 shots of 32 nl each of dsRNA or water (control) once a week for 8 consecutive weeks using a Nanoject Microinjector (Drummond). For the experiment with low-dose irradiation, we also used as control animals injected with *DjOps* dsRNA prepared as previously described [18].

### Whole mount in situ hybridization

WISH was carried out as earlier described [22]. DNA templates for *DjMCM2*, *Djgata4/5/6*, *DjCoe*, *DjNB-21*.*11* and *DjEgr-1* were prepared by RT-PCR as described in Salvetti and coworkers [23], Gambino and coworkers [22], Gambino and coworkers [24], Cassella and coworkers [20] and Gambino and coworkers [7], respectively. *DjMap1B* template was obtained using the following primers:

*DjMap1B* forward
5’ AAGTGATCCTCAAGCTACAGA 3’
*DjMap1B* T7 reverse
5’ CGGATATAATACGACTCACTATAGGGGCATATTCTCAATGTTTGGTAT 3’

PCR products were purified and used for RNA probe synthesis using digoxigenin (DIG)-labelling mix (11277073910, Roche). For each experimental class not less than 10 animals were used and at least 2 independent experiments were performed for each probe. Hybridization signal intensity was measured as mean grey value by Image J software as described in [18].

### Immunofluorescence

Whole-mount immunofluorescence experiments were carried out as described in Gambino et al. [24]. Briefly, samples were fixed and treated like for in situ hybridization and, after hybridization, were incubated with anti-H3P antibody (H3P Ab, 06–570, Sigma‐Aldrich; dilution 1:500), or anti-synapsin (3C11, Developmental Studies Hybridoma Bank; dilution 1:10) or anti-visual-cell antibody, VC-1 [25] (a kindly gift of Dott. Hori; dilution 1:3000). At the end of the procedure, the samples were scanned under a laser scanning confocal microscope TCS SP8 (Leica Microsystems) by zeta stack optical sectioning and tile scan function every 2 μm. Three independent experiments were performed and for each experiment 5 animals were analysed. The counting of H3P-positive cells was performed by using Image J software (find maxima function).

### Morphometric analysis of blastema size

Planarians were amputated below the eyes, producing head (tail regenerating), and tail (head‐regenerating) fragments (Fig 2A). Animals were killed in 2N hydrochloric acid, fixed in 4% formalin and analyzed using a stereomicroscope (Stemi 305, Carl Zeiss Microscopy GmbH), and a camera (Axiocam Erc 5 s, Carl Zeiss Microscopy GmbH). Image J software was used to analyze the digital images. Total body area and the blastema were quantified in 15 animals for each experimental class. The non-pigmented region below the wound epithelium was considered as blastema and its border was manually identified by the operator blindly.

### BrdU-labelling

Animals were injected with 5 shots of 32 nL each of BrdU dissolved in 10% DMSO at a concentration of 10 mg/mL using a Nanojet microinjector (Drummond). Five independent samples (each of 3 animals) for experimental class were treated in 2% HCl in 5/8 Holtfreter for 5 min at 4°C, 24 hours after BrdU injection. Animals were then dissociated in single cells by using 1:1:13 parts of glycerol, acetic acid and dH_2_O for 24 hours at 4°C. BrdU detection was performed as previously described [24] using anti-BrdU antibody (Anti-BrdU B44, BD, 1:50 dilution) in 10% FBS for 1 hour. After washes, slides were incubated with 1:200 goat anti-mouse Alexa Fluor 488 in 10% FBS for 30 min and then mounted in 80% glycerol containing the nuclear dye Hoechst 33342 (Molecular Probes). BrdU- and Hoechst 33342-positive nuclei were counted using a Axioplan microscope (Carl Zeiss Microscopy GmbH). Three independent experiments were performed.

### Flow cytometry analysis

For flow cytometry analysis we followed the ACME fixation/dissociation procedure described by Garcia-Castro and coworkers [26]. Five independent samples were processed for each experimental class. Fixed/dissociated cells were suspended in PBS-BSA plus 0.1% triton X-100 for 15 minutes. Cells were collected at 1200 rpm for 5 minutes and suspended in PBS-BSA containing anti-pS10H3 FLUO antibody (06-570-AF488, Merck, EMD Millipore Corp., 1: 50 dilution) for 2 hours at room temperature. After two washes with PBS-BSA, RNA was digested with 50 μg/mL DNAse-free RNAse A (EN0531, Invitrogen by Thermo Fisher Scientific) for 30 minutes at room temperature and DNA was for 45 minutes in the dark with 1mg/mL PI (P1304MP, Invitrogen by Thermo Fisher Scientific). Stained cells were visualized using an ACCURI C6 PLUS (BD Biosciences) cytofluorimeter. Doublets discrimination was manually performed by plotting events on a linear scale according to FSC signal area vs FSC signal height. Gated events were then visualized in linear scale according to FL3 670 LP channel signal area vs FL3 670 LP channel signal height, in this way a second gating was produced to eliminate debris with very low PI staining a minimum of 25000 gated events were recorded and plotted in FL3 670 LP channel (linear) vs FL1 533/30 channel (logarithmic).

### TUNEL assay

Planarians were treated with 2% HCl in 5/8 Holtfreter solution and then processed as previously described [20] by using ApopTag Red in situ Apoptosis Detection kit (S7165, Merck EMD Millipore Corp). Five planarians for each experimental condition were observed under the Leica TCS SP8 confocal microscope (Leica Microsystems) and three independent experiments were performed. ImageJ software [27] was used to quantify the number of apoptotic cells by find maxima option.

### Transmission electron microscopy

Animals were fixed 2.5% glutaraldehyde in 0.1 M cacodylate buffer pH 7.2 for 2 hours at 4°C and then post-fixed in 0.1 M osmium tetroxide for 2 hours at room temperature as previously described by Salvetti and coworkers [28]. After dehydration by a series of ethanol, samples were embedded in epoxidic resin. Uranyl acetate and lead citrate were used to stain ultrathin sections. The samples were analyzed under a 100 SX electron microscope (Jeol).

### Photophobic assay

The photophobic assay was carried out as previously described by Degl’Innocenti and coworkers [29]. Animals were adapted in the dark for 1 hour before the experiments. A black colored box (length 7 cm, wide 3 cm) filled with planarian artificial water (6 mL) was used. The box was subdivided into 4 sectors and above the first sector a cyano light (emission 480 nm) was put. Animals were dropped between the second and third sector and the number of animals present in the darker sector (the fourth) was counted after 1 and 2 minutes the planarians were placed in the box. Three independent experiments were done and 10 animals for experimental class were used.

### Statistical analysis

The software GraphPad Prism 7.00 was utilized to accomplish a statistical analysis of experiments. Student’s t-test for unpaired data was used to assess the statistical significance of obtained data (p<0.05).

## Results

### *DjMap1B* expression in planarians

**Fig 1:**
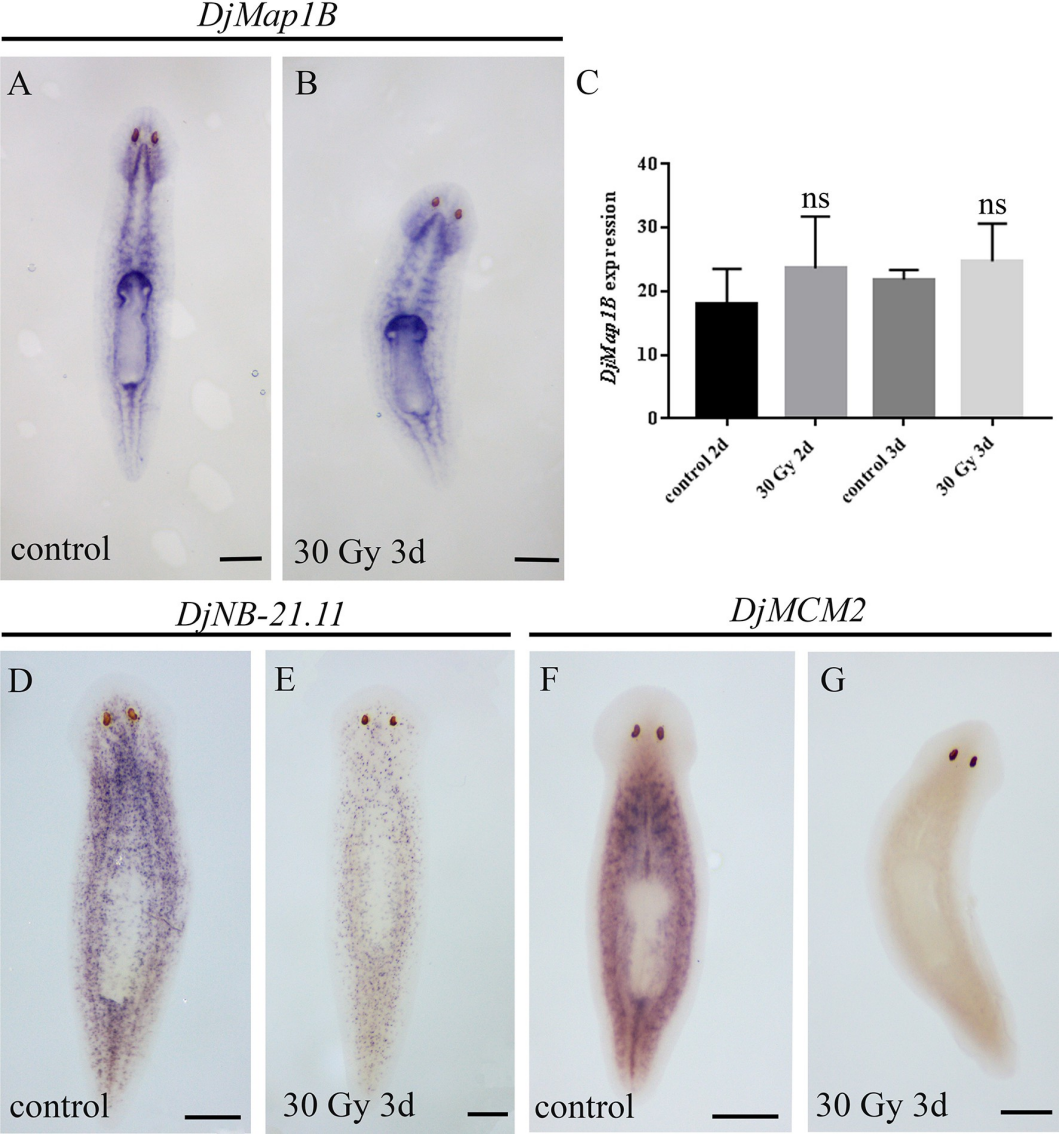
*DjMap1B*, *DjNB-21*.*11* and *DjMCM2* expression in intact planarians. Representative images of *DjMap1B* expression in (A) a control animal and (B) a 30 Gy irradiated animal 3 days after the treatment. (A-B) are ventral views. (C) Graph depicting the quantification of *DjMap1B* expression level. Each bar represents the mean value ± standard deviation of the mean gray values quantified in ten independent samples of a representative experiment. d (days). ns: not significant (p>0.05). (D) Representative images of *DjNB-21*.*11* expression in a control animal and (E) 30 Gy irradiated animal 3 days after treatment. *DjNB-21*.*11* is an early stem cell progeny marker and its expression is reduced after X-ray treatment. (F) Representative images of *DjMCM2* expression in a control animal and (G) 30 Gy irradiated animal 3 days after treatment. *DjMCM2* is a neoblast marker and its expression is strongly reduced very soon after irradiation. (D-G) are dorsal views. Scale bar is 500 μm in A, B, D-G.

By comparing the transcriptional profile of X-ray low-dose treated animals versus untreated controls, we found several up-regulated genes [18] and, among them, we selected *DjMap1B* showing sequence homology with mouse Microtubule-associated protein 1B (e value: 7e-20), *Drosophila* futsch (e value: 7e-27) and *Schmidtea mediterranea* Map1B (*SmedMap1B*, e value: 0). *SmedMap1B* appears mainly expressed in neural, cathepsin positive (i.e. pigment cells, glia, and several other cells with unknown function) and muscle clusters as well as in the neural progenitor subcluster among the *Smedwi-1-*positive cells (https://digiworm.wi.mit.edu/) [30]. *DjMap1B* expression pattern in *D*. *japonica* follows that described in the *S*. *mediterranea* digiworm database, with an appreciable enrichment in the nervous system, i.e. cephalic ganglia and ventral nerve cords in untreated animals (Fig 1A). A similar expression pattern was found in low-dose irradiated animals (S1A and S1B Fig), thus excluding that the overexpression we found by RNA-seq [18] occurs as a consequence of the activation of *DjMap1B* expression in additional planarian cell types. Importantly, the complex *DjMap1B* expression pattern was not affected following lethal (30 Gy) X-ray treatment (Fig 1B and 1C), a condition known to selectively eliminate neoblasts and committed progenies (Fig 1D–1G) but not differentiated cells [23].

### *DjMap1B* role in neoblast biology

**Fig 2:**
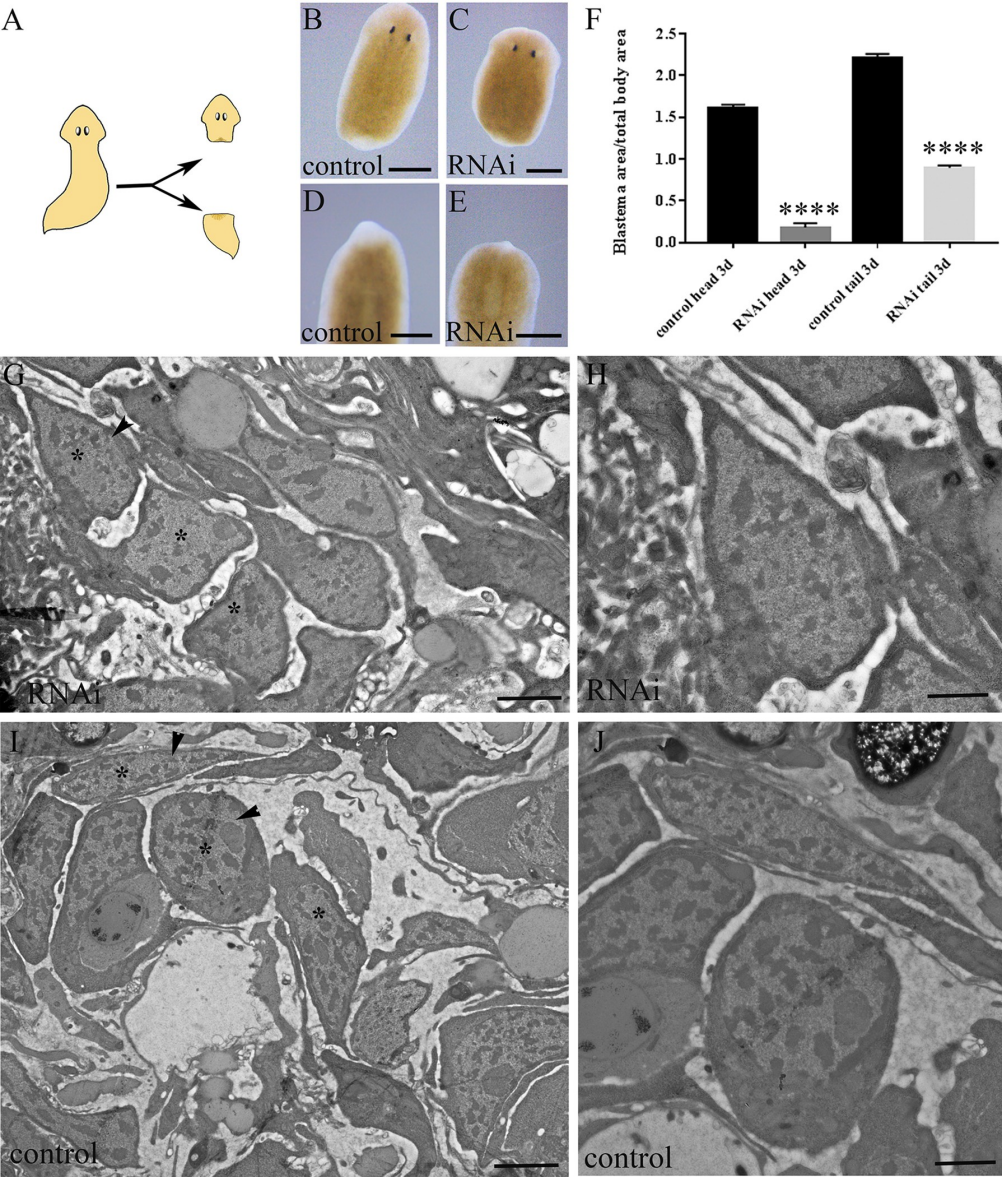
Analysis of regenerating blastema in *DjMap1B*-silenced animals. (A) Schematic drawing of the amputation level producing a head fragment and a tail fragment. (B) A representative head fragment of a control animal and (C) RNAi animal 3 days after amputation. (D) Representative tail fragment of a control animal and (E) RNAi animal 3 days after amputation. (B-E) are dorsal view. (F) Graph depicting the quantification of blastema size. Each bar is the mean ± standard deviation of 15 independent samples of a representative experiment. ****p<0.0001. (G) Representative electron micrograph of the regenerating area of a *DjMap1B-*silenced (RNAi) animal and (I) a control 3 days after amputation showing some neoblasts (asterisks). (H, J) Magnification of the cells indicated by the black arrowheads in G and I showing morphological features of neoblasts. d (days). Scale bar is 500 μm in A-D, 2 μm in G and I, and 800 nm in H and J.

In order to verify *DjMap1B* involvement in neoblast repopulation in animals irradiated with low-dose of X-rays [18], we studied the neoblast marker *DjMCM2* expression [19] in 7 Gy treated animals where we previously silenced *DjMap1B* expression by RNAi. We found a significant reduction in *DjMCM2* expression level in low-dose irradiated animals silenced for *DjMap1B* with respect to low-dose irradiated control and low-dose irradiated animals silenced for *DjOps*, a gene expressed in photoreceptor neurons and not related with stem cells [31] (S1C–S1F Fig), suggesting a role for *DjMap1B* in controlling neoblast activity. Confident with the obtained result, we decided to analyze *DjMap1B* role in physiological conditions such as tissue regeneration, and we found that RNAi animals showed a blastema with a significantly reduced size 3 days after the cut (Fig 2A–2F). Although we always observed this regeneration delay, the extent of reduction in blastema size showed an inter-experimental variability and this delay was usually recovered in about 2 weeks.

**Fig 3:**
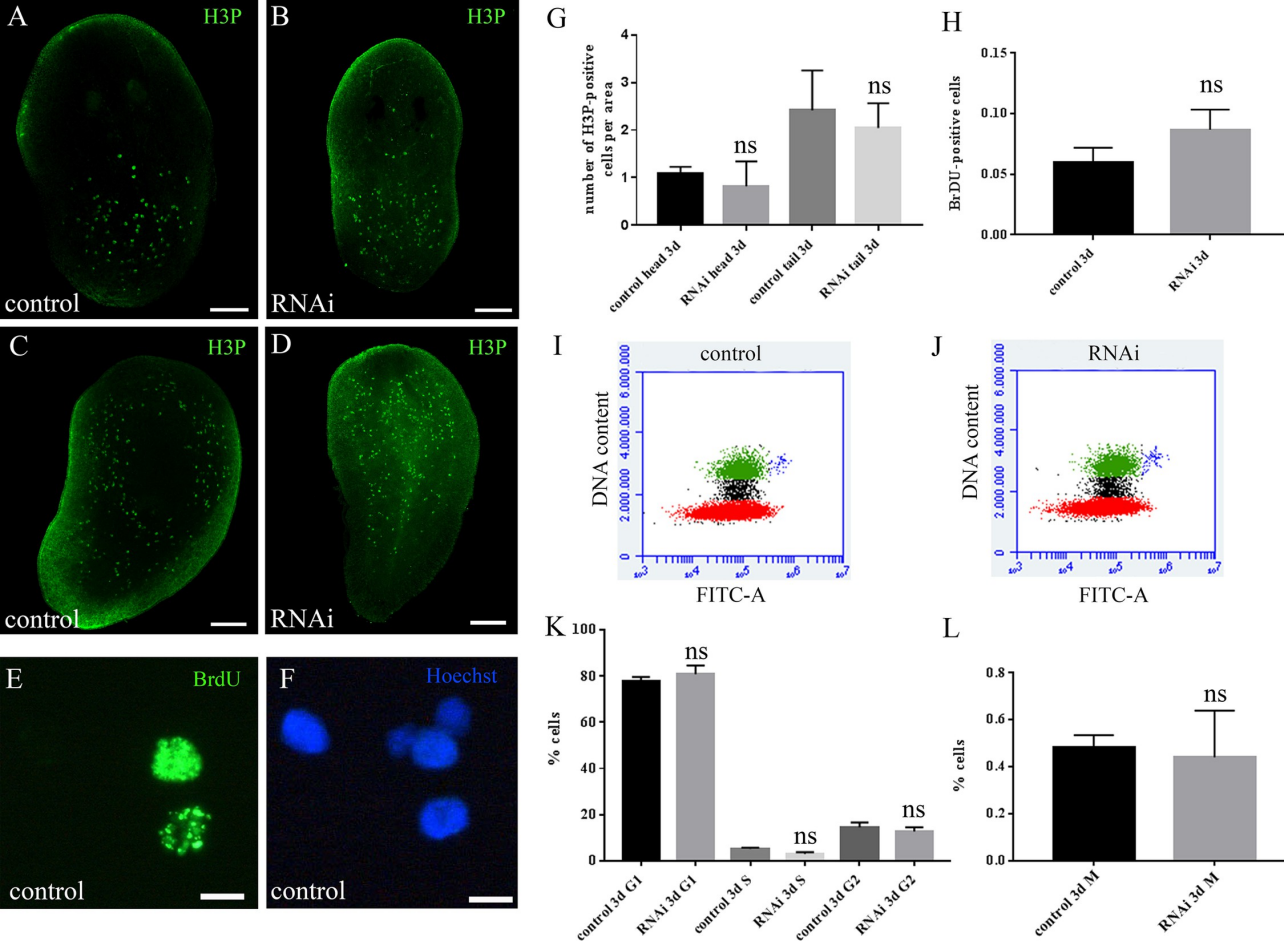
Analysis of proliferating cells in *DjMap1B*-silenced animals. (A) Representative confocal image of H3P-positive cells (green dots) in a control regenerating head fragment and (B) in a *DjMap1B* silenced regenerating head fragment (RNAi) 3 days after amputation. (C) Representative confocal image of H3P-positive cells (green dots) in a control regenerating tail fragment and (D) *DjMap1B* silenced regenerating tail fragment (RNAi) 3 days after amputation. (E) Representative image depicting BrdU-positive cells 3 days after amputation and 1 day after BrdU injection. (F). The same cells in E were stained with Hoechst 33352 blue nuclear staining. (G) Graph depicting the quantification of H3P-expressing cells in control and RNAi regenerating fragments 3 days after amputation. Each bar is the mean ± standard deviation of 5 independent samples of a representative experiment, ns: not significant (p>0.05). (H) Graph illustrating the quantification of BrdU-expressing cells 3 days after amputation and 1 day after BrdU injection. Each bar is the mean ± standard deviation of 5 independent samples of a representative experiment, ns: not significant (p>0.05). (I) Representative plot depicting the distribution in the cell cycle of cells obtained from control animals and (J) *DjMap1B*-silenced animals (RNAi) 3 days after amputation. Red: G1-phase cells; black: S-phase cells; green: G2-phase cells; blue: M-phase cells. (K-L) Graph depicting the percentage of cells in the different phases of the cell cycle. Each bar is the mean ± standard deviation of 5 independent samples of a representative experiment. ns: not significant (p>0.05). d (days); h (hours). Scale bar is 500 μm in A-D and 10 μm in G.

Ultrastructural investigations of the regenerating region revealed the presence of cells with the morphological feature typical of neoblasts (i.e. small cells with high nucleo/cytoplasm ratio, and undifferentiated cytoplasm showing ribosomes and a few mitochondria) in the wound region of RNAi animals in a similar number to what observed in control animals (Fig 2G–2J). With the aim to understand whether neoblasts were able to proliferate in *DjMap1B* silenced animals, we measured the number of mitotic cells by H3P immunostaining, and we found that, despite the reduced blastema size, the number of H3P-positive cells and their spatial distribution were comparable in RNAi animals and controls (Fig 3A–3D and 3G).

Moreover, no differences were found in the number of BrdU-positive cells (Fig 3E, 3F and 3H). These data were also confirmed by flow cytometry analysis of cell cycle with concomitant immunostaining of mitotic cells (Fig 3I–3L).

**Fig 4:**
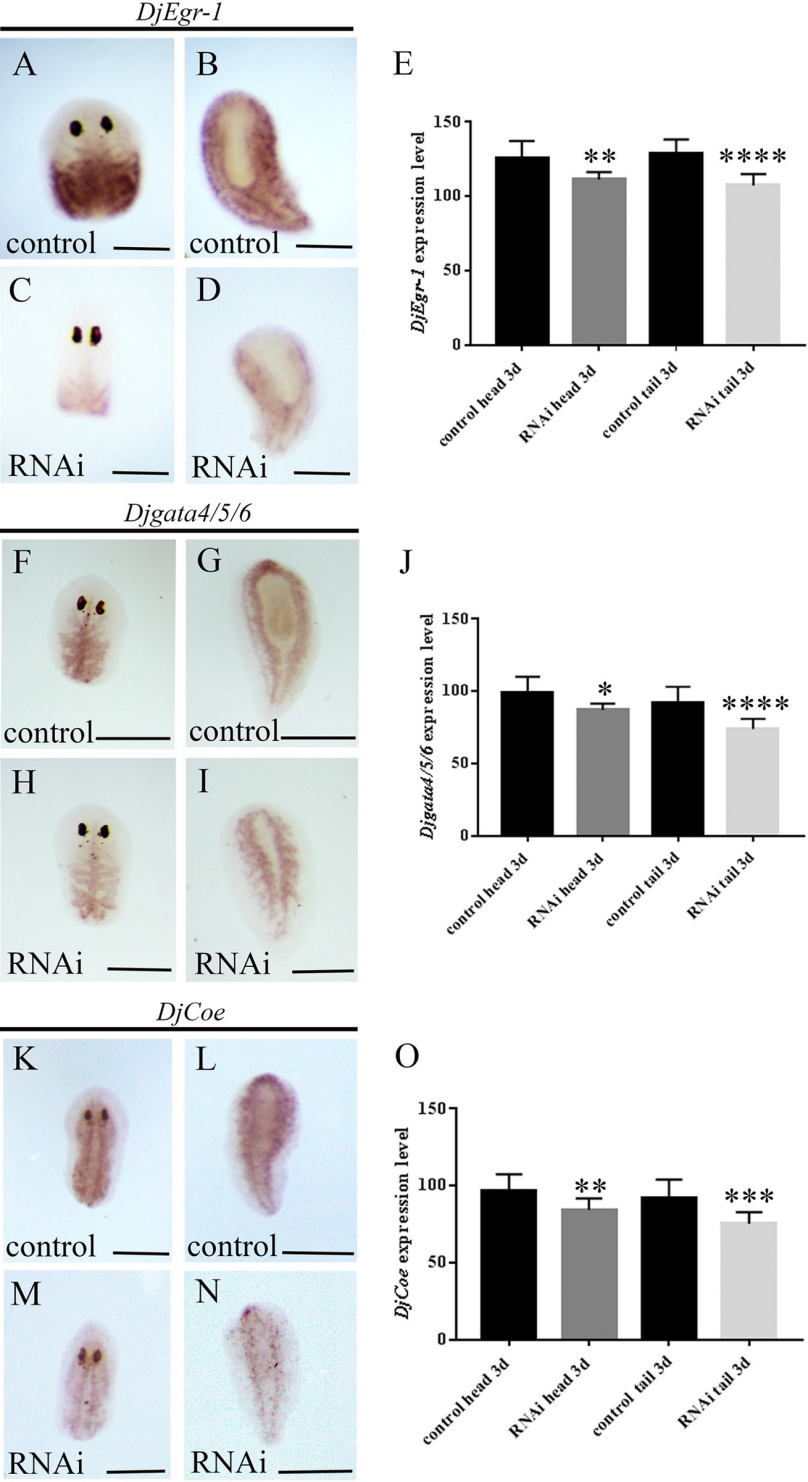
Analysis of marker expression for committed neoblasts in *DjMap1B* silenced animals by WISH 3 days after amputation. (A) Representative images of *DjEgr-1* expression in a regenerating control head and (B) tail fragment. (C) Representative images of *DjEgr-1* expression in a regenerating *DjMap1B*-silenced animals (RNAi) head and (D) tail fragment. (A-D) are dorsal view. (E) Graph depicting the quantification of *DjEgr-1* expression level. (F) Representative images of *Djgata4/5/6* expression in a regenerating control head and (G) tail fragment. (H) Representative images of *Djgata4/5/6* expression in a regenerating *DjMap1B*-silenced animals (RNAi) head and (I) tail fragment. (F-I) are dorsal view. (J) Graph depicting the quantification of *Djgata4/5/6* expression level. (K) Representative images of *DjCoe* expression in regenerating control head and (L) tail fragment. (M) Representative images of *DjCoe* expression in regenerating *DjMap1B*-silenced animals (RNAi) head and (N) tail fragment. (K-N) are dorsal view. (O) Graph depicting the quantification of *DjCoe* expression level. Each bar (E, J, O) represents the mean value ± standard deviation of the mean gray values quantified in 10 independent samples of a representative experiment. Scale bar is 500 μm in A-D, F-I, K-N. *p<0.05; **p<0.005; ***p<0.0005; ****p< 0.0001.

As blastema is formed by the accumulation of committed neoblasts, we analyzed the presence of these cells by using *DjEgr-1*, *Djgata4/5/6* and *DjCoe* molecular markers of epidermal, intestinal and nervous system committed neoblasts, respectively [32–34]. As shown in Fig 4, the expression level of all the selected markers was reduced in RNAi animals with respect to controls.

**Fig 5:**
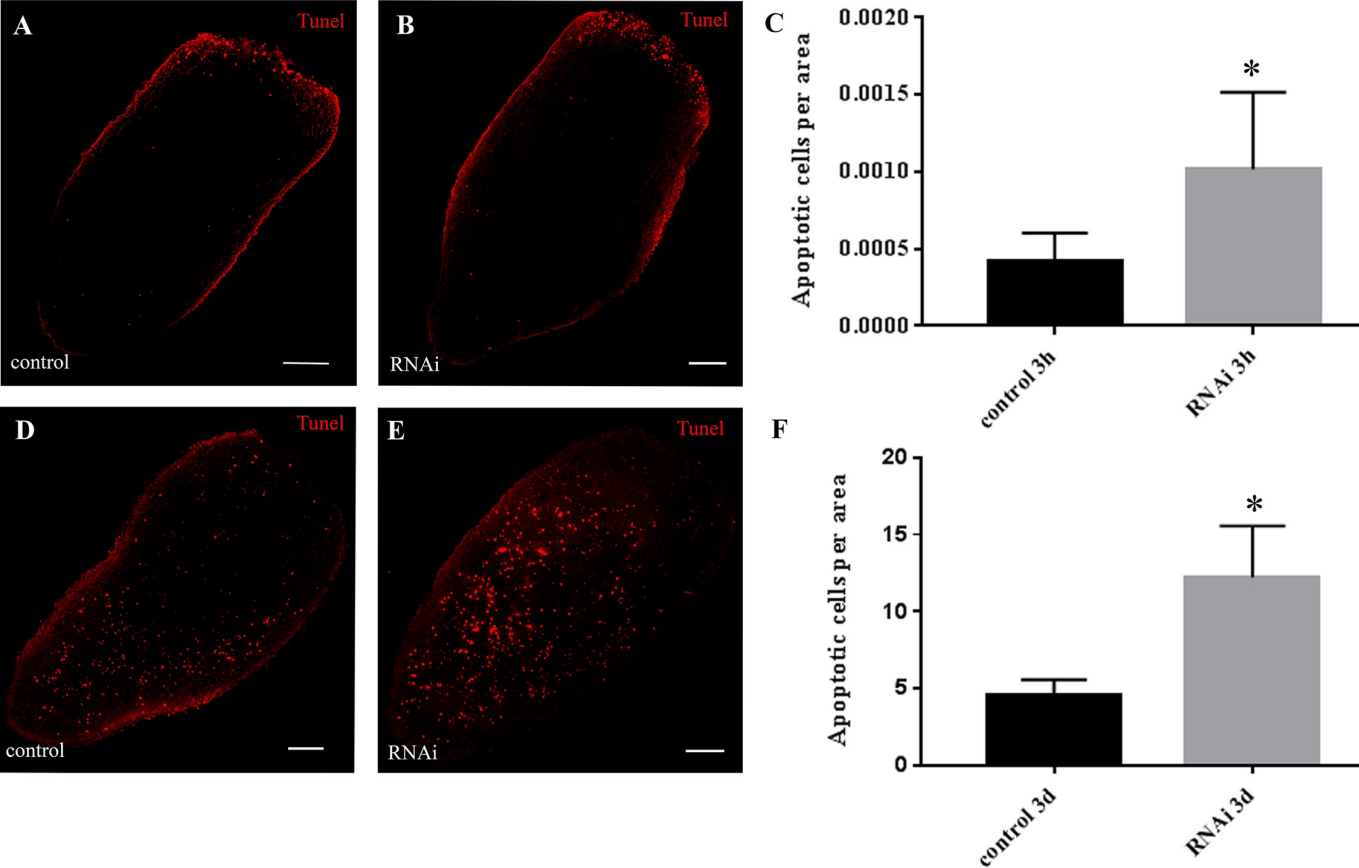
Analysis of apoptosis, by TUNEL assay, in *DjMap1B* silenced animals. In A, B, C data were collected 3 hours after cutting, a time at which apoptotic cells (red dots) are accumulated below the wound epithelium. (A) A representative confocal image of a regenerating control tail fragment. (B) A representative confocal image of a regenerating RNAi tail fragment. (C) Graph depicting the quantification of the number of apoptotic cells. In D, E, F data were collected 3 days after cutting, a time at which apoptotic cells are scattered throughout the parenchyma. (D) A representative confocal image of a regenerating control tail fragment showing cells in apoptosis (red dots). (E) A representative confocal image of a regenerating RNAi tail fragment. (F) Graph depicting the quantification of the number of apoptotic cells. Each bar (C and F) is the mean ± standard deviation of 5 independent samples of a representative experiment. h (hours); d (days). *p<0.05. Scale bar is 100 μm in A-D.

Finally, we studied the biphasic apoptotic pattern [35] by Tunel assay and we found a significant increase of apoptotic cells at both 3 hours as well as at 3 days after amputation in animals silenced for the expression of *DjMap1B* (Fig 5).

### *DjMap1B* role in nervous system

**Fig 6:**
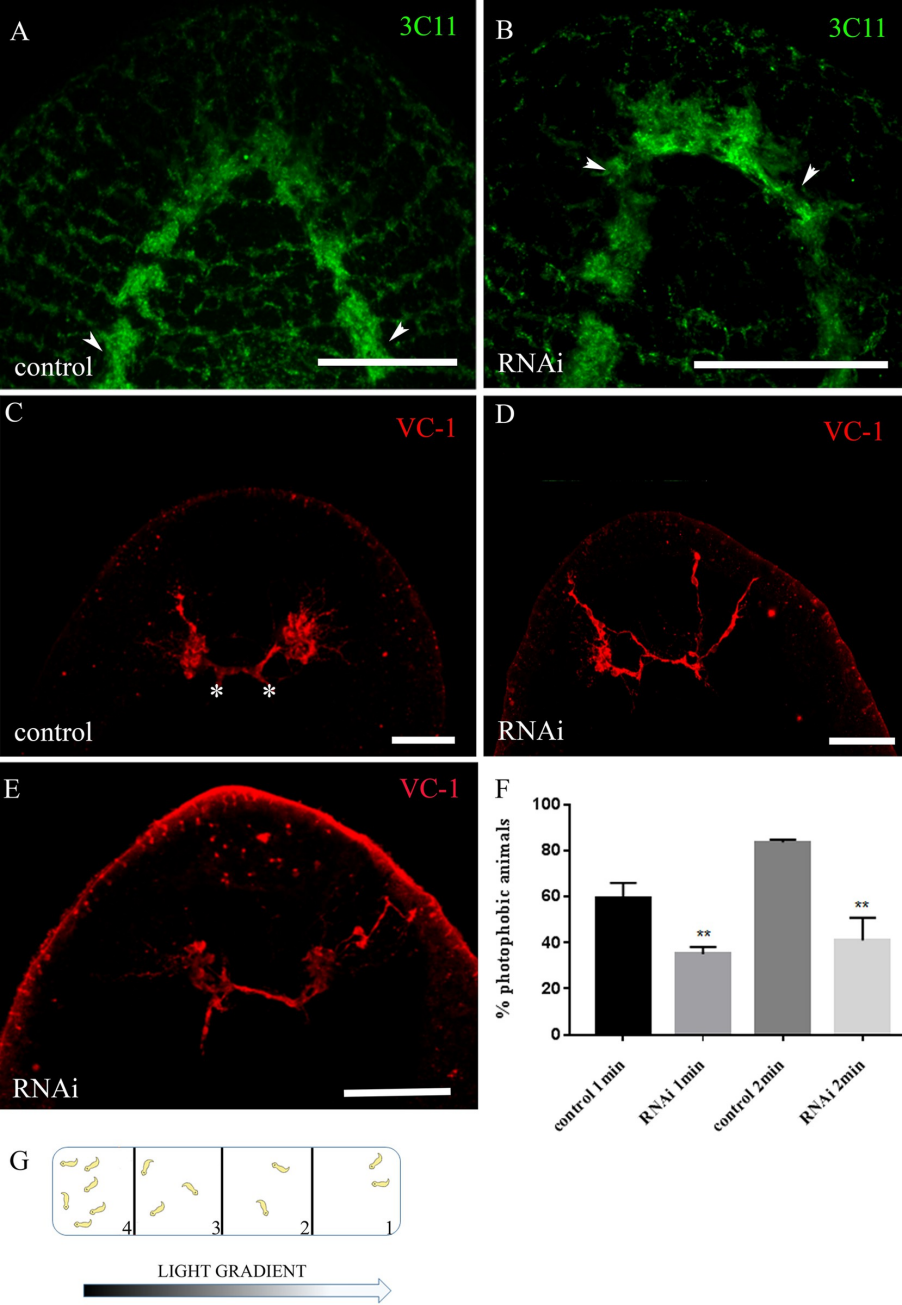
Analysis of the nervous system in *DjMap1B*-silenced animals. (A) Representative confocal images of 3C11 expression in a control head fragment and (B) *DjMap1B*-silenced (RNAi) head fragment 7 days after amputation. Cephalic ganglia with U-inverted shapes are indicated by arrowheads. (C) Representative confocal images of VC-1 distribution in a control tail fragment and (D, E) in *DjMap1B* silenced (RNAi) tail fragment 7 days after amputation. Asterisks indicate the medial region of the brain where visual neurons projected from the optic chiasm. (F) Graph depicting the percentage of photophobic intact animals when exposed to cyano light. Each bar is the mean ± standard deviation of 10 independent samples of a representative experiment. min (minutes). Scale bar is 25 μm in A, B and E and 50 μm in C, D. (G) Drawing of the experimental chamber used for behavioral experiments, with cyano light scattering from the right side. The brightest quadrant is number 1; the darkest quadrant is number 4. **p<0.005.

As *DjMap1B* is expressed at the brain and nerve cord level, we analyzed the regeneration of nervous system by using 3C11, the pan neural marker against synapsin, 7 days after amputation (Fig 6).

Animals silenced for the expression of *DjMap1B* showed reduced cephalic ganglia with an irregular shape (Fig 6A and 6B). Moreover, RNAi animals also showed anomalies at the level of the optic chiasm where in some cases (12/15) ectopic visual axons were observed that projected towards the epidermis and formed loops (Fig 6D and 6E), instead of projecting towards the central part of the cephalic ganglia (Fig 6C). Abnormal optic chiasms were also detected after 16 days after cutting. Although we failed to detect morphological anomalies in the nervous system of RNAi intact animals, silenced animals showed reduced ability to move in response to light compared to control animals (Fig 6F and 6G).

## Discussion

Understanding the molecular and cellular mechanisms that allow a stem cell to self-renew or to undergo a differentiation process is essential to allow the application of stem cells in the biomedical field. Planarian regeneration includes blastema formation via stem cell proliferation, determination and differentiation thus offering an exceptional opportunity to study *in vivo* stem cells.

*DjMap1B*, encoding for a putative protein homolog to MAP1B factors that are involved in the organization, stabilization and function of the cytoskeleton [36], was originally identified among transcripts differentially regulated during the neoblast repopulation process following sub-lethal irradiation [18]. In way to verify its possible function in the repopulation process, here we demonstrate that *DjMap1B* silencing significantly slows down the accumulation of *DjMCM2*-positive cells in the animal body following low-dose X-ray treatment. Strikingly, by analyzing the expression pattern of *DjMap1B*, we find out that its transcripts are enriched in the central nervous system in untreated as well as in animals exposed to low-dose irradiation, and no *DjMap1B* was detectable in neoblasts. Indeed, no reduction in the level of *DjMap1B* expression occurs when the animals are treated with a lethal dose of X-rays, a treatment known to eliminate the neoblasts as well as committed progenies.

The expression of *DjMap1B* in the nervous system is consistent with the expression of other MAP1B homologs. Three members of MAP1 family have been described (MAP1A, MAP1B, MAP1S) with different expression patterns and, among them, MAP1B factors are specifically located at the nervous system level where they are expressed in neurite and growing axons and are fundamental for brain development as well as neuroarchitecture maintenance [37, 38]. The fact that silencing of a gene expressed in differentiated cells affects neoblast behavior is particularly intriguing suggesting a possible role for *DjMap1B* in the non-cell autonomous control of the neoblast system. Accordingly, *DjMap1B* is increased in low-dose irradiated animals but not in lethal irradiated animals, similarly to what happens to other genes involved in neoblast repopulation [18], thus suggesting that the enrichment in *DjMap1B* expression is not a consequence of X-ray exposition but rather a specific activation to coordinate stem cell dynamics by crosstalk between remaining neoblasts and differentiated cells.

This possibility pushed us to investigate the function of *DjMap1B* in physiological conditions, i.e. tissue regeneration. Animals silenced for *DjMap1B* experience a regeneration delay showing a reduction in the size of the regenerative blastema that might be explained by a reduction in neoblast proliferation and /or an increase in apoptosis. When we analyzed the cutting region of RNAi animals by electron microscopy, we found the presence of accumulated neoblasts and we also found that the number of S-phase and M-phase cells was comparable to that of controls indicating that animals silenced for *DjMap1B* possess proliferating neoblasts. On the contrary, regenerating silenced animals showed an increase in apoptotic cells. We don’t know which cells die, but we found a reduction in the expression of committed neoblast markers. So, a possibility is that the silencing of *DjMap1B* could affect the commitment of neoblasts, thus inducing their death and, consequently, a regeneration delay, being the blastema formed by the accumulation of committed neoblasts originating from proliferating neoblasts located in the underlying wound region.

So, a possible functional link between DjMAP1B activity in neurons and its effects at the neoblast level might reside in the fundamental role of MAP1B factors in controlling cytoskeleton function in constituting a pathway to the cell membrane for secretory vesicles during exocytosis processes. In this context, a functional network between microfilaments and microtubules has been proposed [39]. MAP1Bs have been found essential for integrity, function and regeneration of axons [36], and they have been implicated in functional coupling actin filaments and microtubules [40]. It has been proposed that they act as scaffold proteins between cytoskeleton and membranes, probably by functionally linking different proteins [41]. The *Drosophila* MAP1B homolog, *Futsch*, has an important role in stabilizing the active zone where it regulates the release of neurotransmitters [42, 43], and in neuronal retention in adult flies [44]; Futsch mutants have impaired axon regeneration [45]. So it is possible to hypothesize that also *DjMap1B*, like the other members of MAP1B family, is involved in neuroarchitecture maintenance and neurosecretion throughout an action at the level of the cytoskeleton filaments present in the nerve cell terminal. The silencing of this gene could therefore lead to morphological alterations at the nervous system level and modifications of the physiological release of neurosecretory materials involved in the exogenous control of neoblasts. To support this possibility, we found that *DjMap1B* silencing caused alterations at the nervous system and optic chiasm level as well as behavioral defects in response to light, indicating a modification in the nervous system functionality of these animals and according to the fact that neural networks are implicated in controlling phototaxis [46]. Recently, it has been demonstrated that *notum*^*+*^;*frizzled 5/8-4*^+^ muscle cells together with a neuronal population promote visual system regeneration acting as guidepost-like cells [47]. Further studies will be necessary to understand whether DjMap1B interferes with these mechanisms. Neoblast non-autonomous control by the nervous system have been already proposed in planarians. Peptides belonging to tachykinin family, as substance P and K, are potent mitogen for neoblasts [9], and somatostatin inhibits cellular proliferation [14] thus supporting the possibility that neoblasts have receptors for neuropeptides on their cell membrane. Moreover, an insulin-like peptide has been found expressed in cephalic ganglia and nerve cords and it has been demonstrated that impaired insulin/IGF signaling affects neoblast proliferation thus suggesting also the presence of neuroendocrine receptors on neoblasts [11].

In conclusion, data obtained further suggest a role of the nervous system in planarian stem cell biology and put forward a correlation of a MAP1B factor with stem cells, thus paving the way for further studies in higher organisms in order to increase the understanding of the mechanisms that regulate the maintenance and determination of stem cells.

## Supporting information

S1 Fig *DjMap1B* expression and role in low-dose irradiated animals. Representative images of *DjMap1B* expression in (A) a control and (B) 7 Gy irradiated animal 7 days after irradiation. (A-B) are ventral views. (C) Graph depicting the quantification of *DjMCM2* expression level in 7 Gy irradiated animals (control 7 Gy) and in 7 Gy irradiated animals silenced for the expression of *DjMap1B* (dsMap 7 Gy) or *DjOps* (dsOps 7 Gy). Each bar represents the mean value ± standard deviation of the mean gray values quantified in 10 independent samples of a representative experiment. **p<0.005. (D) *DjMCM2* expression in a 7 Gy irradiated animal and (E) in a 7 Gy irradiated animal silenced for the expression of *DjMap1B* and (F) *DjOps* 7 days after irradiation. (D-F) are ventral views. Scale bar is 500 μm in A, B, D-F.
(TIF)

## Abbreviations

MAP1B: MAP1B Microtubule-associated 1B
RNAi: RNA interference
WISH: whole mount in situ hybridization
Gy: Gray
PI: propidium iodide
H3P: phospho-histone H3
