# Supplementary figures and images for: Microtubule-associated protein 1B is implicated in stem cell commitment and nervous system regeneration in planarians
Source: PLoS One. 2022 Dec 12;17(12):e0278966. doi: 10.1371/journal.pone.0278966 (PMC9744283; doi:10.1371/journal.pone.0278966)

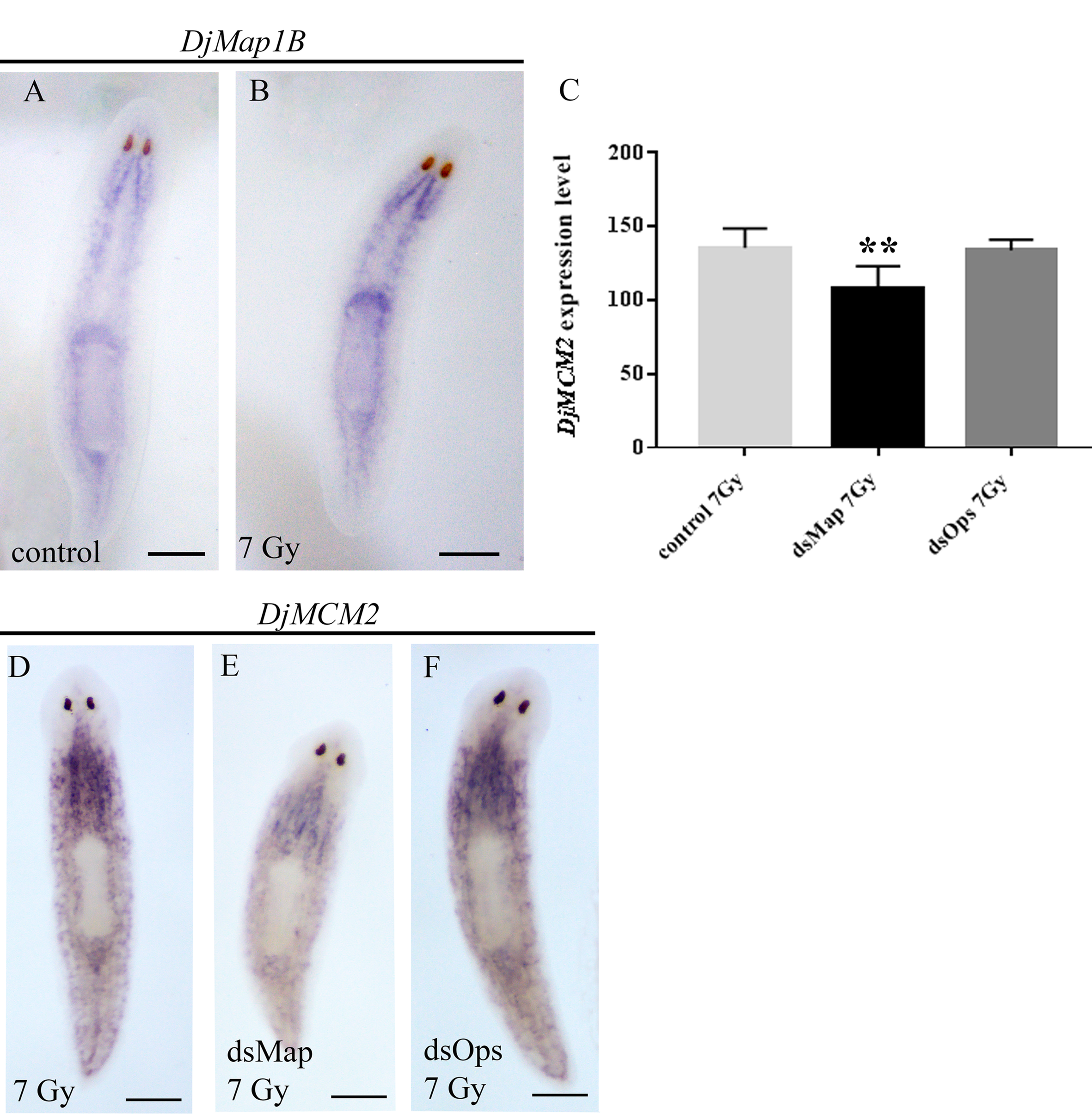

Supplement: S1 Fig — Representative images of DjMap1B expression in (A) a control and (B) 7 Gy irradiated animal 7 days after irradiation. (A-B) are ventral views. (C) Graph depicting the quantification of DjMCM2 expression level in 7 Gy irradiated animals (control 7 Gy) and in 7 Gy irradiated animals silenced for the expression of DjMap1B (dsMap 7 Gy) or DjOps (dsOps 7 Gy). Each bar represents the mean value ± standard deviation of the mean gray values quantified in 10 independent samples of a representative experiment. **p<0.005. (D) DjMCM2 expression in a 7 Gy irradiated animal and (E) in a 7 Gy irradiated animal silenced for the expression of DjMap1B and (F) DjOps 7 days after irradiation. (D-F) are ventral views. Scale bar is 500 μm in A, B, D-F. (TIF) [file pone.0278966.s001.tif]
